# Utilization of Point-of-Care Ultrasound in a Specialist Palliative Care Team Across Multiple Care Settings: A Retrospective Chart Review

**DOI:** 10.1089/pmr.2021.0067

**Published:** 2022-10-21

**Authors:** Amit Arya, Roddy Davey, Achal Sharma, Naheed Dosani, Dilnoor Grewal, Aysha Afzal, Ravi Bhargava, Martin Chasen, Pall Med

**Affiliations:** ^1^ Freeman Centre for the Advancement of Palliative Care, North York General Hospital, Toronto, Ontario, Canada.; ^2^ Division of Supportive and Palliative Care, Brampton Civic Hospital, Brampton, Ontario, Canada.; ^3^ Department of Family & Community Medicine, St. Michael's Hospital, Toronto, Ontario, Canada.; ^4^ Division of Palliative Care, Department of Family and Community Medicine, University of Toronto, Toronto, Ontario, Canada.; ^5^ Division of Palliative Care, Department of Family Medicine, McMaster University, Hamilton, Ontario, Canada.; ^6^ Corporate Department of Research, William Osler Health System, Brampton, Ontario, Canada.

**Keywords:** palliative care, POCUS, symptoms, technology

## Abstract

**Background:**

Technological advancements have rapidly increased the use of point-of-care ultrasound (POCUS) across various medical disciplines, leading to real-time information for clinicians at the bed side. However, literature reveals scant evidence of POCUS use in palliative care. The objective of this study was to examine the use of POCUS in a specialist palliative care setting.

**Methods:**

A retrospective chart review was conducted from January 2018 to June 2019 in Brampton, Canada, to evaluate characteristics of patients for whom POCUS was utilized. Patients were identified through pre-existing logs and descriptive information was collected from electronic health records, including demographic information, life-limiting diagnosis, patient assessment location, diagnosis made with POCUS, and, if applicable, volume of fluid drained.

**Results:**

We identified 126 uses of POCUS in 89 unique patients. Sixty-two patients (69.7%) had a cancer diagnosis, with patients most commonly suffering from gastrointestinal, lung, and breast pathologies. Sixty-one POCUS cases (48.4%) were in the outpatient setting. Eighty-one POCUS cases (64.3%) revealed a diagnosis of ascites and 21 POCUS cases (16.7%) revealed a diagnosis of pleural effusion. Other diagnoses made with POCUS included bowel obstruction, pneumonia, and congestive heart failure. During the study period, 52 paracentesis and 7 thoracentesis procedures were performed using POCUS guidance.

**Conclusion:**

We identified multiple indications in our specialist palliative care setting where POCUS aided in diagnosis/management of patients in both inpatient and outpatient settings. Further studies can be conducted to identify the potential benefits in symptom burden, patient and caregiver satisfaction, and health care utilization in palliative care patients receiving POCUS.

## Introduction

The delivery of palliative care is constantly evolving to better suit the needs of patients. Palliative care is delivered in a multitude of different settings by interprofessional teams consisting of palliative care physicians, nurses, physiotherapists, social workers, and other allied health staff.^1–3^ Specialist palliative care has grown rapidly, and provides more flexibility in provision of care to these patients based on their individual needs. This has resulted in better symptom control, patient satisfaction, improved quality of life and reduced visits to the hospital, and a decreased number of in-hospital deaths.^4^

At the same time, over the past several decades, there has been rapid growth of use of point-of-care ultrasound (POCUS) for physicians in critical care medicine and emergency medicine.^5^ This diagnostic tool allows clinicians to obtain real-time visualization and rapid results right at the patient's bedside with reduced procedural complications.^5,6^ Current literature shows the extent of POCUS use in diagnosing conditions such as malignant and nonmalignant ascites and pleural effusions to assess the need for bedside procedures such as paracentesis and thoracentesis.^7^ POCUS can detect the presence of ascites with a high degree of accuracy and also has superior sensitivity and specificity for locating pleural effusions, when compared with physical examination or chest radiography.^8,9^

POCUS has been shown to improve physician confidence^10^ and has been associated with significantly fewer adverse events such as postdrainage infection and hematoma. Furthermore, recent advances in POCUS technology have made it more accessible to patients in the outpatient setting through portable compact devices that continue to become less expensive, and require minimal infrastructural support.^11^

Patients receiving palliative care often develop complications such as ascites or pleural effusions, which cause debilitating symptoms such as pain, dyspnea, loss of appetite, swelling, and reduction in an individual's mobility, interfering with their quality of life.^6^ In fact, respiratory or abdominal complaints were listed in the top 10 reasons for patient visits to the emergency during the past 30 days of their lives.^12^ Despite the prevalence and advantages of the use of POCUS, and the frequency of indications where POCUS would be beneficial, there is scarcity of evidence examining POCUS use in palliative medicine.

The aim of this retrospective chart review was to explore the utilization of POCUS in a specialist palliative care setting. The study characterized the profiles of patients in which POCUS was utilized and examined the different applications of POCUS in both inpatients and outpatients.

## Materials and Methods

This retrospective chart review was conducted on all palliative care patients in whom POCUS was utilized. All patients were receiving care from palliative care physicians working at William Osler Health System (WOHS), a large multispecialty community hospital in Ontario, Canada. The standard of practice before using POCUS at this hospital involved referring patients to the radiology team. Our clinical experience was that having patients wait for assessment and intervention from the radiology team resulted in delayed relief of symptoms due to increased wait times. As a result, POCUS, which can provide immediate results at the bedside, was introduced as a modality.

In January of 2018, the Division of Palliative Care at WOHS's Brampton Civic Hospital purchased two portable POCUS units for use: (1) a Clarius C3 black and white scanner with virtual phased array and (2) a Sonosite iViz ultrasound system. Two of our physicians undertook prior training in ultrasonography and served as mentors for other colleagues in the division. The palliative care physicians who were responsible for conducting POCUS assessments on the patients in this study obtained their training through various accredited, continuing medical education courses and workshops, including a preconference workshop presented before the Advanced Learning in Palliative Medicine Annual Conference, and McMaster University Simulation Based Learning programs.

Investigators were also provided with on-site clinical mentorship and supervision by senior experienced physicians within the division. Physicians conducting POCUS assessments kept logs of patients in whom POCUS was indicated between January 2018 and June 2019 inclusive, and these patients were reviewed for the scope of the study. Information retrieved included demographic information (age, gender), life-limiting diagnosis, location of patient assessment, diagnosis made with POCUS, and, if applicable, volume of fluid drained. All other relevant information was collected from electronic health records and stored on secure Microsoft spreadsheets.

Institutional ethics approval was granted by the William Osler Research Ethics Board at WOHSs (REB no. 18-0043). Local research ethics board review was conducted and approval was received before any research activity. Owing to the nature of this research study being a retrospective chart review, obtaining explicit informed consent was not required. There was minimal risk to the subjects and did not adversely influence the rights and welfare of the subjects.

## Results

A total of 89 unique patients were identified. Some patients received POCUS on multiple occasions, resulting in 126 total assessments. Of the 89 patients, 53% were females (47) and 62% were >70 years old (View Table 1). Sixty-seven patients (75%) had a cancer diagnosis, and one of the patients was diagnosed with two primary malignancies. Gastrointestinal cancers accounted for 46% of all cancer diagnoses. Nine patients (10%) in our cohort had multiple diagnoses using POCUS.

**Table 1.** Patient Demographics

Demographics	Patients
	*n* = 89
Gender distribution	Females
	*n* = 47 (52.81%)
Patients per age group	30–39—1 (1.12%) 40–49—5 (5.6%) 50–59—18 (20.2%) 60–69—10 (11.2%) 70–79—25 (28.1%) 80–89—26 (29.2%) 90–99—4 (4.5%)
Type of cancer	
Gastrointestinal^*^	45.6%
Lung	19.1%
GU^**^	19.1%
Breast	10.3%
Unknown primary	2.9%
Plasma cell	1.5%
MDS	1.5%
Diagnosis	*n* (%)
Ascites	81 (59.5)
Pleural effusions	21 (15.4)
Pneumonia	13 (9.6)
CHF	2 (1.5)
Bowel obstruction	2 (1.5)
Pneumothorax	1 (0.8)
No diagnosis	16 (11.8)
Location of POCUS uses	*n* (%)
Palliative care unit	57 (45)
Clinic	30 (24)
Home	22 (17)
Long-term care centers	7 (6)
Other locations	10 (8)
Procedures	*n*
Paracentesis	52
Thoracentesis	7
No intervention	67

CHF, congestive heart failure; GU, genitourinary; MDS, myelodysplastic syndrome; POCUS, point-of-care ultrasound.

The assessments using POCUS were distributed across admitted patients (67, 53%) and patients in the outpatient setting (59, 47%). The 126 assessments conducted using POCUS yielded 120 diagnoses (Table 1) and 59 procedures. Table 2 summarizes the breakdown of the total number of procedures conducted. POCUS assessments also yielded diagnoses of pneumonia, congestive heart failure, bowel obstruction, and pneumothorax in descending order. An average of 2.55 L of fluid was drained during all paracentesis procedures, ranging from 0.6 to 6.8 L. An average of 1.14 L fluid was drained from all patients who had a thoracentesis, which ranged from 0.5 to 1.8 L. Two paracentesis procedures resulted in no fluid being drained.

**Table 2.** Patients with Diagnosis of Pleural Effusion and Ascites

		Drainage procedure performed	Drainage procedure not indicated
Diagnosis of pleural effusion (*n*)	21	7	14
Diagnosis of ascites (*n*)	81	52	29

## Discussion

Overall, there is scarcity of data on the utility of POCUS in palliative medicine. In one of the first few reports in the literature concerning POCUS use in palliative care, Gishen et al. described the use of POCUS in an inpatient unit.^13^ The authors reported drainage of ascites as the most common use of POCUS, in addition to other indications. To date, there have been only a few small studies that demonstrated use of POCUS in outpatient palliative care settings.^10,14–17^

These smaller studies report on successful assessments using POCUS in hospice settings, as well as preventing unnecessary procedures and trips to the hospital.^18–20^ One retrospective chart review reported on patients with ascites in nonhospital settings such as hospice, residential care, and patient homes.^10^ The most prevalent pathology in the cohort was ovarian cancer, followed by various gastrointentinal cancers, lung cancers, breast cancers, genitourinary cancers, and cancers of unknown origin. In our study, which included 89 patients, we found a similar distribution of patients across all cancer types (Table 1).

Our retrospective study is the first of its kind to measure the utilization of POCUS in a comprehensive specialist palliative care program that provides patient care across multiple care settings, including homes, long-term care facilities, and outpatient clinics in addition to hospital-based support.^4^ Furthermore, the study highlights the opportunities of POCUS use for a variety of diagnoses, with assessment of peritoneal or pleural fluid being the most common indication.^10,21,22^ Specifically, POCUS has long been established as a tool to help clinicians distinguish between fluid accumulations causing symptomatic sequelae in patients and other pathological abnormalities. We notably observed that 47% of our patients received bed-side interventions assisted by POCUS.

Fifty-three percent of patients in our cohort did not require fluid removal, likely due to inadequate amount of fluid present, assessed using POCUS. This is in line with what has been observed in other studies as well. Landers et al. reported that 19 of 32 patients (59%) had fluid accumulation that was removed through POCUS.^7^ They also reported another patient wherein loculated fluid was observed during POCUS assessment, but was not removed due to inaccessibility. Dhamija et al. mention that POCUS assessments can help clinicians differentiate abdominal distension due to fluids as compared with other causes, thus reducing unnecessary procedures, which could put the patient at risk of complications such as bowel perforation.^5^

Pneumonia is another established cause of morbidity and mortality in patients with advanced life-limiting diagnosis, and is associated with increased discomfort.^23,24^ Considering the different pulmonary causes of distress in palliative care patients, it becomes imperative to effectively diagnose these issues to facilitate competent care, and specifically to facilitate discussion around goals of care. POCUS utility has been well documented in differentiating lung pathologies in critically ill patients, including pneumonia, pneumothorax, pulmonary embolism, and obstructive respiratory disorders.^25^

However, use of POCUS for diagnosis of pulmonary complexities has been fairly limited in palliative care. We were effectively able to diagnose 13 cases of pneumonia and one pneumothorax, further signifying the utility of POCUS across multiple indications. Using POCUS, we were able to diagnose patients with congestive heart failure exacerbation and bowel obstruction as well, which expands the indications for POCUS. Gishen et al. also explored additional indications in their patients, which shed further light on POCUS utility.^13^

There is insufficient evidence in the literature with regard to POCUS assessments in multiple different sites in the same patient. However, we observed this to be the clinical picture in 10% of our patients, where multiple symptoms led to the use of POCUS to diagnose or rule out different etiologies in an individual. Our study adds to the literature by reporting on this unique aspect of the utility of POCUS in such scenarios.

### Limitations

This study was associated with limitations. First, the data collected in our study did not look at adverse events associated with POCUS-guided procedures. Second, the study did not collect health utilization data that would help highlight the cost effectiveness of administering POCUS in the community, an area that can be looked at in future studies. Third, the study was focused on a single site and may not be reproducible at other sites. Although many patients had other imaging per clinical standard of care, diagnoses made by POCUS were not routinely confirmed with other imaging modalities.

These data can, therefore, not comment on the sensitivity or specificity of POCUS in our cohort. Owing to the descriptive nature of this study, we did not identify explicit evidence that POCUS interventions helped decrease hospitalizations. This limitation could be incorporated into future study designs to help answer an important question of quantifiable benefit of using POCUS. Finally, the study focused on the outcomes of the use of POCUS for trained palliative care physicians but did not highlight the processes used to provide this training to clinicians.

## Conclusion

Through our retrospective chart review of palliative care patients on whom POCUS was utilized, POCUS appears to be a versatile tool that can assist the physician in a variety of palliative care settings, both for aiding with diagnosis and for guiding procedures. In particular, the most common uses of POCUS in our specialist palliative care setting are for diagnosis and management of ascites and pleural effusions in patients with advanced cancer. Considering symptoms caused by these fluid collections contribute significantly to hospital visits for patients near the end of life, POCUS provides great potential for efficient and patient-centered care. Future studies on POCUS use in a palliative care setting may look to further measure patient symptom control and satisfaction, while also assessing health care utilization and cost.

## Clinical Palliative Care Program

The research study was conducted by the Division of Supportive and Palliative Care at WOHS, a not-for-profit health care organization consisting of three community hospitals, including Brampton Civic Hospital, Etobicoke General Hospital, and Peel Memorial Centre. The Division of Supportive and Palliative Care provides specialist palliative care in multiple settings, including an outpatient clinic at WOHS, patients admitted at these hospitals, long-term care centers, hospice support, home care services, specialized support for the structurally vulnerable populations, and virtual palliative care as well. The team consists of 10 specialist palliative care physicians, along with a palliative clinic manager and an allied health team of 5 nurses, 0.1 FTE spiritual worker, and 0.2 FTE social worker. In the fiscal year 2019/20, the team realized 1,694 clinic visits, 2028 home visits, 2871 inpatient consults, and 1742 discharges.

## Abbreviations Used

CHF: congestive heart failure
GU: genitourinary
MDS: myelodysplastic syndrome;
POCUS: point-of-care ultrasound
WOHS: William Osler Health System
